# Digital health interventions for spinal surgery patients: A systematic scoping review

**DOI:** 10.1177/20552076251328549

**Published:** 2025-04-15

**Authors:** Annemieke Y van der Horst, Saskia M Kelders, Ernst T Bohlmeijer, Karlein M G Schreurs, Jan S Jukema

**Affiliations:** 1 Research group Smart Health, 2975 Saxion University of Applied Sciences, Enschede, the Netherlands; 2 Department of Psychology, Health and Technology, Centre for eHealth & Wellbeing Research, 3230 University of Twente, Enschede, the Netherlands; 3 Research group Learning to flourish, Fontys University of Applied Sciences, Eindhoven, the Netherlands; 4 Optentia Research Focus Area, 56405 North-West University, Vanderbijlpark, South Africa; 5 Roessingh Research & Development, Enschede, the Netherlands; * Current affiliation: Dimence Mental Health Group, Zwolle, the Netherlands; Saxion Research & Graduate School, Saxion University of Applied Sciences, Enschede, the Netherlands.

**Keywords:** eHealth, mHealth, digital health intervention, digital behavior change intervention, spinal surgery

## Abstract

**Introduction:**

The potential of digital health interventions to optimize healthcare is promising also in the context of spinal surgery. However, a systematic review assessing the quality of digital health interventions for spinal surgery patients and the potential effects on these patients is lacking.

**Method:**

The objective of the current scoping review was to provide a systematic overview of digital health interventions for spinal surgery patients described in scientific literature. The focus was on describing the current digital health interventions, assessing the quality of these descriptions, reviewing the reported effects and assessing the methodological quality of the included studies.

**Results:**

A total of 14 full-text articles, describing 11 digital health interventions were included in the final analysis. These digital health interventions ranged from a website and app to a mobile phone messaging system and mobile phone interface. Most digital health interventions aim to improve adherence to rehabilitation guidelines and physical health. The included studies were generally of moderate to high quality and showed significant effects on physical health. Vital aspects of digital interventions such as “working mechanism theory” and “prompts and reminders” were often absent in the description of interventions.

**Conclusion:**

The study of digital interventions for spinal surgery patient is emerging and promising. However, there is a scarcity of studies using a rigorous design. A more systematic and comprehensive framework for developing and describing digital interventions for spinal surgery patients is highly recommended.

## Introduction

Digital health interventions are health services delivered electronically through formal or informal care providers.^1^ These interventions are in principle accessible at any time or location, are able to provide personalized medicine, enable patients to self-manage health-related activities, and involve them more in their own treatment.^2^ Both self-management and patients’ involvement in care are important because patient engagement has been repeatedly linked to better health outcomes, such as improved coping skills, self-efficacy and self-management skills.^3,4^ Digital health interventions are becoming more common for patients and healthcare providers around the world, including in the orthopedic field.^5–7^ Specifically in peri-operative care, digital health interventions are broadly applied.^8,9^

In the specific context of surgery, digital health interventions can be used to aid patients during their recovery process.^10^ For instance, these interventions can be used for supporting healthy lifestyle behavior change before and after surgery to improve postoperative outcomes^11,12^ and to improve medication adherence.^13,14^ In addition, digital health interventions can be used to prepare patients for surgery or to shorten postoperative recovery through behavior modification, patient monitoring, or protocol adherence.^15–18^ Behavior modification can also be applied to promote a healthy lifestyle. Healthy lifestyle behavior changes before and after orthopedic surgery, such as increased preoperative physical activity or smoking cessation have been associated with improved postoperative bone healing,^19^ wound healing,^20^ quicker recovery times, and reduced pain scores.^21^ Also, discharge instructions are better adhered to by patients, when supported by digital health interventions.^22^ Moreover, surgical patients who engage with digital health interventions show better medication adherence, greater patient satisfaction, improved clinic attendance, lower readmission, and less emergency department visits after surgery.^23^ In a comprehensive review, Van der Meij et al. (2016) found in most studies that perioperative digital health interventions improved clinical patient-related outcomes compared to only face-to-face perioperative care for patients who have undergone various forms of surgery.^24^ This shows the potential of digital health interventions in improving perioperative care, in addition to face-to-face meetings with professionals.

When discussing digital health interventions for spinal surgery patients, the importance of a multidisciplinary approach in addressing low back pain (LBP) needs to be emphasized. In some cases, the cause of LBP can be easily attributed to a single event or condition, such as degenerative disc disease, facet joint syndrome, or spondylolisthesis. However, chronic LBP is often the result of a complex interaction of clinical, psychological, surgical, and traumatic factors.^25^ According to the biopsychosocial model, LBP is best understood through the interaction of physical, psychological, and social influences.^26^ Given the complexity of chronic LBP, a multidisciplinary assessment is imperative and does indeed show promise in targeting LBP.^27,28^ In clinical practice, various specialists, including general practitioners, orthopedists, pain therapists, psychiatrists, and others, contribute to the management of LBP. Each of these healthcare professionals brings distinct objectives and expertise to the treatment process. Therefore, an effective digital health intervention for spinal surgery patients must consider this multidisciplinary approach, although integrating diverse information and addressing the varied needs and interests of different stakeholders can present challenges.

There is a wide range of different types of digital health interventions, including websites and apps that require a smartphone. Recent research shows that orthopedic patients are open to using a smartphone during their patient journey. Reinecke et al. (2021) surveyed 1055 orthopedic surgery patients of which 89,57% indicated they owned a smartphone.^29^ The majority of these patients obtained medical information via mobile web access and were willing to pay for a medical app.^29^ Nathan et al. (2020) conducted a survey in 2017, showing that 75% of spinal surgery patients would be interested in using a digital health intervention to communicate with their care providers and to track their post-operative progress.^30^ In sum, the potential of digital health interventions seems promising and the majority of orthopedic, including spinal surgery, patients is willing to use this type of interventions.

However, an underlying theoretical framework or sound scientific evidence for the effectiveness of digital health interventions is often lacking.^31,32^ Scientific research on the effectiveness of digital health interventions should include a focus on the theoretical foundation and understanding of the working mechanisms of these interventions, to improve their development, efficacy, uptake, dissemination, and evaluation. The design of digital interventions should be anchored in behavioral change theories to optimally engage patients in the intervention and the behavior change.^33,34^ In a review of 85 studies employing the Behavior Change Support Systems of Oinas-Kukkonen & Harjumaa (2009), less than half referred to theories of behavior change, but those that did were uniformly successful.^35^ Clearly, a sound theoretical base for digital health interventions is warranted for optimal behavioral change and effectiveness.

In addition to using a theoretical framework to substantiate digital health interventions, the interventions in scientific studies should be described in a detailed manner as suggested by Eysenbach et al. (2011). Providing a detailed description of researched digital health interventions, clarifies generalizability and transferability of these interventions and enables future researchers to expand on this body of knowledge. The CONSORT eHealth Checklist was developed, specifically for the description of eHealth interventions.^36^

Several attempts have been made to provide an overview of digital health interventions, including mHealth applications, for spinal surgery patients. Bai et al. (2020) performed a narrative review on mHealth applications specifically for spinal surgery patients, as well as applications for other target populations.^6^ Yet, these authors did not follow PRISMA or other relevant guidelines. In a similar way, Goz et al. (2019) outlined several mHealth applications that could potentially be applied to spinal surgery patients.^31^ They stated that there remains a paucity of data on behavior modification and preoperative patient optimization using mHealth tools in the spine population and that development of spine-specific applications and further study of the effectiveness of mHealth application in spine populations is needed.^31^ Chen et al. (2019) performed a review on smartphone applications in orthopedic surgery but focused on capabilities such as angular management, preoperative templating, and quantification of gait instead of behavioral change.^37^ Kolcun et al. (2020) aimed to examine the current utilization of telemedicine for spine surgery but did not focus on behavior change in their review.^38^

In sum, a systematic overview of scientific literature for digital health interventions specifically for spinal surgery patients, including a clear description of the content of the interventions and methodological quality of studies into these interventions, is lacking. By conducting a systematic search of the current scientific literature, we aim to provide healthcare researchers and professionals with an overview of available digital health interventions and the quality of these interventions. This enables professionals to expand and improve the care for their patients. In addition, providing an overview of digital health interventions shows researchers where possibilities lie for future research, e.g. targeting specific patient populations or using more theoretical frameworks in the development of digital health interventions. To this purpose, the objective of the current scoping review is threefold: to provide an overview of digital health interventions for spinal surgery patients described in scientific studies, including whether they use a theoretical framework (1), to assess the quality of the intervention descriptions in these studies (2) as well as the reported effects of the studies (3).

## Material and methods

### Research questions

1. Which digital health interventions for spinal surgery patients are described in scientific literature?
2. What is the quality of the intervention description in these studies?
3. What effects are reported and what is the methodological quality of these studies?

These research questions are addressed using the PRISMA guidelines^39^ (RQ1); the CONSORT eHealth checklist by Eysenbach^36^ (RQ2) and a checklist by Hawker et al.^40^ (RQ3).

### Scoping review methodology

As technology evolves rapidly, a scoping review methodology was chosen as this technique provides a timely overview of the current literature on this topic. As the aim of the review was to include all digital health interventions for spinal surgery patients reported in scientific literature, not only these tested in RCTs, a scoping methodology was most fitting for this review. This review displays the quantity and quality of available literature sorted by study design and other key features, which fits a scoping review methodology.^41^ As PROSPERO does not accept scoping reviews, this review is not registered.

### Information source

With Scopus, Pubmed, and Web of Science, we aim to cover a wide range of relevant references. By adding the specialized databases of PsycINFO, CINAHL, and IEEE Xplore we aimed to add unique references matching the scope of our review covering psychology, nursing, and technology.

The search in all databases was performed on 3 May 2023.

### Search

For the full search strategies, see the Appendix. In the searches, no restrictions on the type of article or publication period have been used, to ensure a broad range of results. Synonyms for all main concepts were used: e.g. digital health intervention and digital behavior change intervention for different types of interventions; surgery and operation for the medical procedure; spinal and lumbar for the specific type of surgery.

### Eligibility criteria

In this review, the term “digital behavior change intervention”^42^ is used to specify which interventions to include in the search. Also, in this review, technology should be the carrier of the intervention. Technology should not (only) be the means or facilitator by which the patient and professional communicate. This for instance excludes the use of technology to facilitate telemedicine through video-conferencing between patient and professional, without any other form of intervention.

In Table 1, the eligibility criteria are described, stating which inclusion and exclusion criteria were used during the selection process. Only studies written in English were included.

**Table 1.** Eligibility criteria for included studies.

Category	Inclusion criteria	Exclusion criteria
*Condition or domain*	Spinal surgery patients	Spina bifida; spinal cord injury; scoliosis
*Patient population for intervention*	Spinal (or synonym), surgery/operation has to be mentioned in title and/or abstract n.b.: no exclusion criteria for age of the population	Target population is (medical) professionals Target population is other (surgery) patients Target population is spinal cord injury, spina bifida or scoliosis patients (too specific needs)
*Intervention*	Article describes one or several digital health application(s) or intervention(s) that have been used by patients Intervention described must be digital (i.e. technological) Intervention described must be an intervention with an active participation of the patient with the aim to change the behavior of the participant (i.e. not telemedicine with only consultation as aim) Any focus/aim for the intervention, include all aims	Intervention has not been used or tested yet (e.g., design or protocol article) Technology which is not an intervention (e.g., for physical monitoring, to administer pain medication or electronic health records) Technology only used for (physical) examination, diagnosis or communication (e.g., telemedicine) Technology only used for physical outcomes (e.g., 6 min gait test, posture or angle measurement) Technology used for computer assisted surgery or telerobotic surgery Article focusses on judging You Tube videos Article focusses on online surgeon ratings by patients
*Control group*	The use of a control group is possible, but not obligatory for a study to be included in the review.	
*Study design and language*	Original article (thus no systematic reviews on several studies) n.b.: no exclusion criteria for date/year, all years are included	Not an original article (e.g., a systematic review) Title/abstract/full text is in a different language than English Full text not available (not at all or not in English)

**Figure 1.**
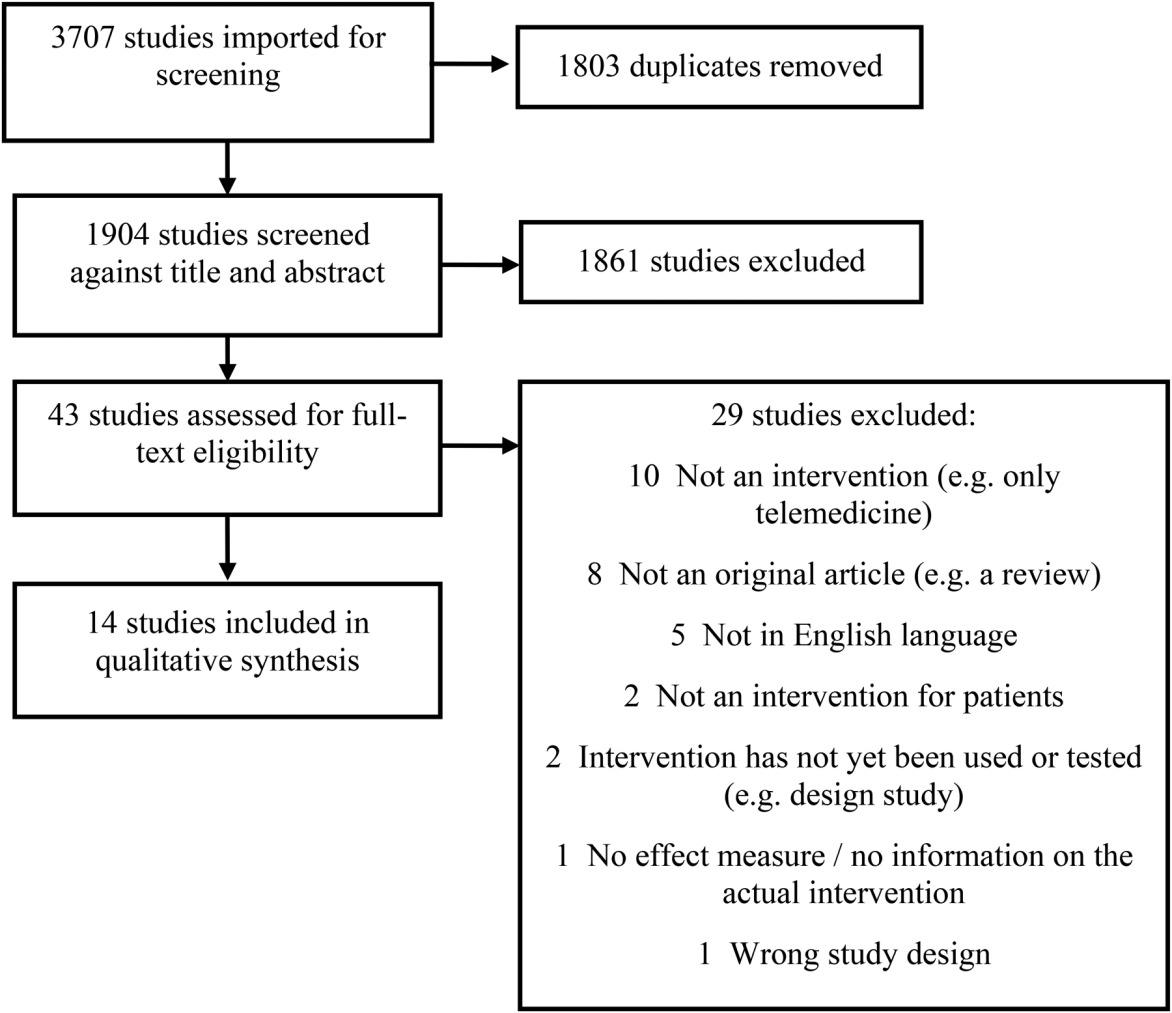
Overview of full search and selection process according to PRISMA statement.

The full search of all selected databases yielded 3707 studies, including 1803 duplicates (see Figure 1).

### Study selection

A selection of retrieved articles based on the title was done independently in Covidence by two of the authors (AvdH and JJ). Abstracts were also independently reviewed (by AvdH and JJ) using Covidence. Discrepancies were discussed, and consensus was reached. After these two, separate steps, full-text selection was performed by the same authors, again independently and a consensus on discrepancies was reached. After excluding irrelevant studies 14 full-text articles were included in the final review.

### Data collection process

Data extraction from the full-text articles (*n* = 14) was done by one author (AvdH) in three ways: by using a data extraction form in Excel; by assessing the description of the digital health interventions using the CONSORT eHealth checklist for evaluation of Web-based and Mobile Health Interventions^36^ and by assessing the methodological rigor of the research, using the quality assessment by Hawker et al.^40^ The results of this data extraction process were reviewed by a second author (JJ) and discussed until consensus was reached.

### Data extraction form

Details that were collected of all included studies in the data extraction form can be divided into two categories: details on the *study* and details on the *intervention* described in the study. Data collected on the *study* included: author, year, and country; study design (e.g. pilot study, RCT); number of study participants; key participant characteristics (e.g. age and gender); outcome measures of the study; and main findings of the study. Data collected on the digital health *interventions* included: name of the digital health intervention, target population; description of the intervention; focus, goal, or aim of the intervention (e.g. education, exercise, monitoring, mental health); moment of use of the intervention (e.g. before and/or after surgery); type of technology (e.g. app), and whether a theoretical framework was used for the intervention.

### Quality assessment

The *description of the digital health intervention* in the included studies was assessed by two authors (AvdH and JJ) using the CONSORT eHealth checklist for evaluation of Web-based and Mobile Health Interventions.^36^ The included studies were rated on all 12 sub-items of the checklist on a 3-item scale: yes (2), partially (1), no (0). Sum scores, formed by adding all sub-items, ranged from 0 to 24.

The *methodological quality* of the included studies was evaluated by two authors (AvdH and JJ) based on a tool developed by Hawker et al.^40^ This tool consists of nine items to assess the methodological rigor of a study: abstract and title; introduction and aims; method and data; sampling; data analysis; ethics and bias; presentation of results; transferability and generalizability; implications and usefulness. Each item was scored either very poor (1), poor (2), fair (3), or good (4), with a possible sum score ranging from 9 to 36.

## Results

### Description of included studies

An overview of the included studies is shown in Table 2. A total of 14 full-text articles was included in the final analysis. These studies describe a total of 11 unique digital health interventions.

**Table 2.** Description of included studies.

	Author, year, country	Study design	No. of study participants	Key participant characteristics	Measures	Main findings
*1.*	Erdogan & Bulut, 2020 Turkey	RCT	Total *N* = 62 Intervention group *n* = 31 Control group n = 31	Intervention group: 14 women, 17 men; control group: 13 women, 18 men Average age of 40 (range 20–65)	Level of knowledge; level of anxiety (State-Trait Anxiety Inventory); problems experienced after discharge; and level of functional incapacity (ODI).	Patients in the intervention group *showed higher levels of* *information, higher functional capacity, and reduced anxiety* compared with the control group.
*2.*	Felbaum et al., 2018 United States	Prospective observational study	*n* = 56	33 women, 23 men; median age of 54 (range 27–79)	Compliance with instructions, canceled surgeries, complications and readmissions, and patient satisfaction.	54 of the 56 patients used the app and complied with instructions. No canceled surgeries. One postoperative complication. No readmissions.
*3.*	Gautschi et al., 2010 Switzerland	Prospective study	*n* = 52	20 women, 32 men; mean age 55.06, (range 21–86)	Patient satisfaction with the system	*Positive impact on patient education* derived from *high satisfaction scores* (>82%)
*4.*	Glauser et al., 2019 United States	Prospective evaluation (pilot) study	*n* = 30	30 participants, 22 non-users, 8 users^a^. The 8 users: 5 female, 3 male, age mean 50.88	Patient feedback on accessibility, ease of use and ease of implementation of the app.	Of the non-users^a^ **27.3%** felt the app would *not be helpful and* 36.4% had difficulty registering. Of the users 0% had difficulty registering. The users^a^ found the daily to-do list and the walking measures to be the *most helpful components* of the app.
*5.*	Goz et al., 2019 United States	Pilot study	*n* = 21 (app users, of which 12 were interviewed)	5 women, 7 men; average age 57.8 (range 34–78)	Patient feedback on the app and usage of the app (no. of messages sent)	The tool was *felt to be useful* by nearly all patients, had a *high degree of patient engagement*, and made the majority of patients *less likely to call clinic*.
*6.*	Guo et al., 2022 China	Prospective study	Total *N* = 72 Intervention group *n* = 36 Control group *n* = 36	Intervention group: 23 women, 13 men, average age 57.6 (SD 10.25) Control group: 15 women, 21 men, average age 62.3 (SD 9.58)	Exercise compliance, pain, disability, self-efficacy	The intervention group showed *significant improvement in exercise compliance, pain and self-efficacy* compared to the control group. No significant difference was found in dysfunction improvement.
*7.*	He et al., 2021 China	RCT	Total *N* = 95 Study group *n* = 47 Control group *n* = 48	Study group: 22 women, 25 men, age 46.01 (SD 5.12) Control group: 21 women, 27 men, age 45.88 (SD 4.99)	Performing recommended exercises (compliance), patient's waist activity (surgery effectiveness), quality of life, spinal nerve function, lumbar function.	The results showed that the *compliance rate* (performing recommended exercises) of the study group was 89.36%, *significantly higher* than that of the control group (60.42%). The *effective* *rate* (patient's waist activity restored to normal) of the study group was 95.74%, *significantly higher* than that of the control group (81.25%). Further, continuous nursing based on wechat platform brought *more obvious improvement in quality of life as well as the spinal nerve function and lumbar function.*
*8.*	Hou et al., 2019 China	RCT (multicenter, prospective, randomized controlled trial)	Total *N* = 168 Intervention group *n* = 84 Usual care group *n* = 84	Intervention group: 48 women, 36 men, mean age 51.11; usual care: 42 women, 42 men, mean age 49.36;	Function (ODI); pain (VAS); general mental health; quality of life (EuroQol-5; 36-SFHS).	*Improvement of primary outcomes (pain and function)* in the intervention group was superior to the usual care group at 24 months postoperatively. The effectiveness of eHealth was more evident in participants with higher compliance.
*9.*	Ponder et al., 2020 United States	Development, feasibility and pilot study	*N* = 47	22 women, 25 men, age range 33–77 (mean 59, SD 11).	Back Disability Index, Neck Disability Index, percent pain reduction	Of the 24 patients who completed the MMS survey, 21 (*88%) said it was* *helpful during preparation* for their procedure, 16 *(67%) said it was helpful during the postoperative period,* and 23 *(96%) said* *that they would recommend the app* to a friend or family member.
*10.*	Stewart et al., 2019^b^ United States	Prospective study	Total *N* = 174 Intervention group *n* = 85 Non-app-user group *n* = 89	Intervention group: 43 women, 42 men, average age 53; non-app-user group: 49 women, 40 men, average age 59.7	Compliance with instructions, canceled surgeries	*All 85 app users adhered to preoperative instructions* according to the acknowledgements sent to the web portal, and there were *no cancelled surgeries.* Among the 89 *non-app users,* there were *five cancelled surgeries (*5.6%).
*11.*	Strøm, Hoybye, et al., 2019 Denmark	A prospective cohort with a mixed methods design	*N* = 48	26 women, 22 men; mean age of 53 (range 29–77).	Characteristics of users (gender, age, level of education, employment status, marital status), use of the ISG^/^^c^ (number of posts, comments, interactions, page views), anxiety and depression (HADS), content analysis of the ISG posts and comments.	Sociodemographic characteristics were not predictors of ISG^c^ use in this study, *women were more prone to be active users.*
*12.*	Strøm, Nielsen, et al., 2019 Denmark	A single-center, two-arm, RCT.	Total *N* = 99 Intervention group *n* = 48 Control group *n* = 51	Intervention group: 26 women, 22 men; control group: 38 women, 13 men; mean age of 54.	Anxiety and depression (HADS), pain (LBPRS), disability (ODI), and health-related quality of life (EQ-5D-5L).	*No significant effect of the intervention found on any of the outcome measures.* However, the *high compliance* and degree of interaction with the intervention indicates that it could be applicable in this group of patients.
*13.*	Venkatraman et al., 2022^d^ United Stated	A retrospective study	Total *N* = 1015 App users *n* = 679 Non users *n *= 336	App users: 312 women, 367 men, average age 57.9 (SD 12.5) Non users = 177 women, 159 men, average age 61.5 (SD 12.7)	Rates of 90-day emergency room (ER) visits, readmissions, and complications	After adjustments were made for age and sex, the *odds of having 90-day ER visits for app users were 32% lower than those for nonusers*, but this difference was *not statistically significant* (odds ratio 0.68, 95% CI 0.45–1.02; P = .06).
*14.*	Von Glinski et al., 2020 United States	A retro-spective pilot study	*N* = 55	17 women, 38 men. mean age of 57 (range 25–81)	Number of APDS^e^ uses, change of treatment, readmission and patient satisfaction.	The data suggest that patient *age, gender, or invasiveness* of surgery is *not associated with the usage* of APDS^e^

a Non-users were grouped as participants that logged one or two days of incomplete information pre-operatively, with no post-operative data input. Users were considered as patients with high involvement in the app, having logged in nearly every day from a week pre-op to >45 days post-op.

b Stewart et al., 2019 builds on Felbaum et al. 2018.

c ISG stands for Internet Support Group.

d Vekatraman et al., 2022 builds on Ponder et al., 2020.

e APDS stands for Active Post Discharge Surveillance.

Except for the study of Gautschi et al.,^43^ all included studies were published in 2018 or more recently. Half of the included studies (*n* = 7) was performed in the United States of America,^16,44–49^ three in China,^50–52^ two in Denmark,^53,54^ one in Switzerland,^43^ and one in Turkey.^55^

Seven studies used an intervention group and a control or usual care group,^47,48,50–52,54,55^ of which four studies were randomized controlled trials.^51,52,54,55^ The number of participants in all the included studies ranged from 21 to 1015, with a median of 59. The mean age of participants ranged around the age of 50–55.

Concerning outcome measures, the studies can be divided into one of three categories: studies focusing on patient-reported outcome measures (e.g. functional disability, pain, quality of life); studies focusing on adherence or compliance with recovery and hospital regulations (with outcomes such as readmissions, canceled surgeries and compliance with instructions); and studies focusing on the evaluation of the digital health intervention (e.g. measuring patient satisfaction with the intervention).

### Description of included digital health interventions (RQ1)

In the 14 included studies, a total of 11 different digital health interventions were described. Felbaum et al. (2018) and Stewart et al. (2019) described the same digital health intervention: Track my recovery (Amie). Ponder et al. (2020) and Venkatraman et al. (2022) described the same intervention: Manage My Surgery-spine. Strøm, Hoybye, et al. (2019) described an ISG (Internet Support Group) which is part of the intervention w-SPINA, described in more detail in Strøm, Nielsen, et al. (2019). The characteristics of these 11 digital health interventions are displayed in Table 3.

**Table 3.** Description of included digital health interventions.

	Name and target population	Description and type of technology (bold)	Focus, goal or aim	Moment of use	Theoretical framework
1.	Erdogan & Bulut, 2020 N: Not specified T: Lumbar disc herniation surgery patients	On the *training website*, users were able to navigate the site using training menus, ask questions, access answers to frequently asked questions, complete questionnaires, use a personal message box, and see relayed messages and announcements following from the instructions for use.	To increase uptake of information Lower anxiety Decrease number of problems after discharge Increase level of functional capacity	From day of surgery Duration: not specified	Not specified
2.	Felbaum et al., 2018 N: Track my recovery (Amie) T: Neurosurgery patients	A *smartphone app* designed to provide tailored, surgeon-driven pre- and postoperative instructions. The app also permitted patients to send pain scores and images of their wound at regular intervals while allowing physicians and their staff to send messages to their patients.	To improve compliance with instructions Prevent canceled surgeries Reduce complications and readmissions Improve patient satisfaction with the intervention.	Both before and after surgery Duration: not specified	Not specified
3.	Gautschi et al., 2010 N: Not specified T: Elective lumbar disc surgery patients	*Web-based* audiovisual *information system* with pictures, text and videos, installed on a tablet	To inform patients about surgical procedures in order to amend the conversation between patient and physician before a scheduled operation.	Day before surgery	Not specified
4.	Glauser et al., 2019 N: Neuropath (the Neuropath application) T: Elective spine surgery patients	A patient and provider *mHealth app/smartphone app* (platform) supporting the goals of opioid monitoring and reductions, wound care, enhanced activity after surgery, and enhanced recovery after surgery (ERAS)	To improve care while decreasing costs and postoperative pain.	Both before and after surgery Duration: not specified	Not specified
5.	Goz et al., 2019 N: Not specified T: Spine surgery patients	A *mobile phone messaging* patient education *system* Patients received daily text messages with educational material regarding their recovery	To inform patients of their expected postoperative course with the goal of decreasing patient anxiety.	After surgery: for 14 days after discharge from their operative admission	Not specified
6.	Guo et al., 2022 N: WeChat group named “Post-discharge Rehabilitation Management for Patients with Lumbar Fusion Surgery” T: Lumbar fusion surgery patients	Individualized post-discharge rehabilitation program, including exercise rehabilitation, posture correction education, daily activity education, correct instructions for lumbar brace wearing, and self-management of pain. In the form of a WeChatbased *electronic education manual* with group meetings every fortnight.	To offer an individualized rehabilitation program.	After surgery Duration: not specified	Not specified
7.	He et al., 2021 N: WeChat group named “Postoperative group of patients with LDH” T: Lumbar disc herniation surgery patients	Patients received continuous nursing based on the *WeChat* platform. The WeChat group included doctors, nurses, patients and their families. The platform included functional exercises, texts, pictures and videos by the nurses.	To help patients understand functional exercises. To increase compliance, effectiveness, quality of life and physical functioning.	After surgery Duration: 3 months	Not specified
8.	Hou et al., 2019 N: Not specified T: Lumbar spine surgery patients	Through the *mobile phone–based interface*, patients were able to view the rehabilitation plans made by their physicians and conduct their rehabilitation following the video instructions. In addition, patients could receive daily reports about their exercise and alerts to prompt them to return to this system. They could also communicate with their doctors through this system.	To provide telerehabilitation, to reduce pain-related disability and improve prognosis.	After surgery (start 3 months after surgery)	Not specified
9.	Ponder et al., 2020 N: Manage My Surgery—Spine (MMS) T: Elective spine surgery patients and their caregivers	The app serves as a virtual patient navigator through the various phases of the surgical journey. The app includes patient-reported outcome measures, FAQ’s, notifications and educational multimedia resources.	To educate and prepare patients for the surgery and make shared decisions, leading to lower overall anxiety, increased satisfaction, and increased retention of patients in their digital care pathway.	Both before and after surgery Duration: not specified in this article, see Venkatraman et al., 2022 for details	Behavior change techniques (BCT's), not further elaborated on, were used to facilitate short-term behavior change.
10.	Stewart et al., 2019 N: Track my recovery (Amie) T: Spinal surgery patients	*Smartphone app* (see no. 2)	To improve patient adherence with preoperative instructions and reduce last-minute surgery cancellations (see no. 2 as well)	Both before and after surgery Duration: not specified	Not specified
11.	Strøm, Hoybye, et al., 2019 N: No name of the ISG is mentioned. The ISG is part of w-SPINA T: Lumbar spine fusion surgery patients	The *ISG* (internet support group) comprised a *message board* visible to all participants. Everything written here was visible for all participants. The ISG was embedded on a platform also containing animated information and training instructions, designed to support LSF patients primarily within the first 3 months after LSF.	To increase overall health-related quality of life and to decrease anxiety and depression	Both before and after surgery. 1–5 weeks before surgery until 3–6 months after surgery.	CBT
12.	Strøm, Nielsen, et al., 2019 N: w-SPINA T: Lumbar spine fusion surgery patients	*A web-based platform* containing animated information, an internet support group and a diary.	To decrease symptoms of anxiety and depression, pain, disability, and increase health-related quality of life.	Both before and after surgery. 1–5 weeks before surgery until 3–6 months after surgery.	CBT
13.	Venkatraman et al., 2022 N: Manage My Surgery—Spine (MMS) T: Spine surgery patients	*A perioperative mobile app* including educational and instructional content, FAQ's, notifications, reminders and questionnaires for patient –reported outcome measures.	To educate patients and collect patient-reported outcome measures, as to reduce ER visits, hospital readmissions and postoperative complications.	Both before and after surgery. 2–4 weeks before surgery and surveys continued until 12 months after surgery	Not specified See Ponder et al., 2020 for details
14.	Von Glinski et al., 2020 N: Active Post Discharge Surveillance (APDS) No specific name T: Elective spine surgery patients	*A mobile phone app*, with a secure health care information and exchange platform containing preoperative reminders, general and specified communications, and educational tips and messages	To reduce readmissions as well as “no-show” rates in clinic, to decrease the number of phone calls, and to ultimately increase overall patient satisfaction.	After surgery Duration: not specified	Not specified

The digital health interventions differed in type of technology from a website, app, and web-based information system to a mobile phone messaging system and mobile phone interface. The most frequently used type of technology, i.e., in six studies, was an app.^44–49^

The focus of the digital health interventions differed between mental or physical health, patient education, organizational, patient satisfaction or cost-effectiveness. The majority of the digital health interventions had a physical focus (8 out of the 11 interventions). An example of a (mainly) physical focus was to reduce pain and physical disability, while increasing exercise compliance and self-efficacy for exercise, as in the intervention of Guo et al. (2022). An example of a mental focus was to reduce anxiety or depression, as in the intervention w-SPINA.^54^ An example of an organizational focus was to improve compliance with preoperative instructions and reduce canceled surgeries, as in the intervention Track my recovery, Amie.^44^

Seven digital health interventions could be used before as well as after surgery. Most of these digital health interventions did not specify the exact duration of the intervention. Five digital health interventions were meant to be used specifically after surgery. One digital health intervention should be used before surgery,^43^ as the aim of this intervention was to inform the patient about the surgical procedure and to elicit a conversation between the patient and their healthcare provider.

Only one study mentioned the theoretical model used in the digital health intervention, namely CBT (cognitive behavioral therapy) which was used for the digital health intervention w-SPINA, described by Strøm, Nielsen, et al. (2019) and Strøm, Hoybye, et al. (2019). One study^46^ mentioned using behavior change techniques (BCTs) to facilitate short-term behavior change in the Manage My Surgery-spine intervention, but the way in which this was done was not elaborated on, nor was this mentioned or described in the study on the same intervention by Venkatraman et al. (2022).

### Quality of intervention description in included studies (RQ2)

The full overview of how the included studies score on the CONSORT eHealth Checklist can be found in Table 4. Four sub-items of the CONSORT eHealth checklist for evaluation of Web-based and Mobile Health Interventions are deemed essential^36^ i.e., access; mode of delivery, content of intervention, and theoretical framework; prompts and reminders; co-interventions. Of these essential sub-items, the sub-item “access” is fully described by only four included studies,^44,46,48,50^ and partially for all other included studies. The sub-item “mode of delivery, content of intervention and theoretical framework” is only partially described by all the included studies. The other two essential sub-items “prompts and reminders” and “co-interventions” are described (partially or completely) by the majority of the studies (resp. 12 and 11 of the 14 studies).

**Table 4.** Intervention description according to the Consort eHealth checklist criteria.

	Subitem i	ii	iii	iv	v	vi	vii	viii	ix	x	xi	xii	
	Highly recommended	Highly recommended	Highly recommended	Highly recommended	Highly recommended	Highly recommended	Essential	Essential	Highly recommended	Highly recommended	Essential	Essential	
	Developers, sponsors, and owners	History or development process	Revisions and updating	Quality assurance methods	Source code and/or providing screenshots/screen-capture video, and/or providing flowcharts of the algorithms used.	Digital preservation	Access	Mode of delivery, features/functionalities/components of the intervention and comparator, and the theoretical framework	Use parameters	Level of human involvement	Prompts/reminders used	Co-interventions (including training/support)	Total sum score per study (min.0–max.24)
Erdogan, 2020	Yes 2	No 0	No 0	No 0	No 0	No 0	Partially 1	Partially 1	Partially 1	Yes 2	Yes 2	Yes 2	11
Felbaum, 2018	Yes 2	Partially 1	Partially 1	No 0	Yes 2	Yes 2	Yes 2	Partially 1	Partially 1	Yes 2	Yes 2	Yes 2	18
Gautschi, 2010	Yes 2	Yes 2	No 0	No 0	Yes 2	No 0	Partially 1	Partially 1	Yes 2	Yes 2	Yes 2	Yes 2	16
Glauser, 2019	Yes 2	Partially 1	No 0	No 0	No 0	No 0	Partially 1	Partially 1	Partially 1	Yes 2	Partially 1	No 0	9
Goz, 2019	Partially 1	Partially 1	No 0	No 0	No 0	No 0	Partially 1	Partially 1	Yes 2	Yes 2	Yes 2	No 0	10
Guo, 2020	Partially 1	Partially 1	Partially 1	No 0	Partially 1	No 0	Yes 2	Partially 1	Yes 2	Yes 2	Partially 1	Yes 2	14
He, 2020	Partially 1	No 0	No 0	No 0	No 0	No 0	Partially 1	Partially 1	No 0	Partially 1	No 0	No 0	4
Hou, 2019	Partially 1	Partially 1	No 0	No 0	Partially 1	No 0	Partially 1	Partially 1	Yes 2	Yes 2	Yes 2	Yes 2	13
Ponder, 2020	Yes 2	Yes 2	No 0	No 0	Partially 1	No 0	Yes 2	Partially 1	Partially 1	Partially 1	Partially 1	Partially 1	12
Stewart, 2019	Yes 2	Partially 1	Partially 1	No 0	Partially 1	No 0	Partially 1	Partially 1	Partially 1	Yes 2	Yes 2	Yes 2	14
Strøm, Hoybye, 2019	Partially 1	Partially 1	No 0	No 0	Partially 1	No 0	Partially 1	Partially 1	Yes 2	Yes 2	Yes 2	Yes 2	13
Strøm, Nielsen, 2019b	Partially 1	Partially 1	No 0	No 0	No 0	No 0	Partially 1	Partially 1	Partially 1	Yes 2	No 0	Yes 2	9
Venkatraman, 2022	Yes 2	Partially 1	No 0	No 0	Partially 1	No 0	Yes 2	Partially 1	Partially 1	Partially 1	Partially 1	Partially 1	11
Von Glinski, 2020	Partially 1	Partially 1	No 0	No 0	No 0	No 0	Partially 1	Partially 1	Partially 1	Yes 2	Partially 1	Yes 2	10
Total sum score per sub item (min. 0–max. 28)	21	14	3	0	10	2	18	14	18	25	19	20	

For the highly recommended sub-items, the best scoring sub-item is “level of human involvement,” which is fully described by almost all included studies (11 out of 14 studies) and partially described by the remaining three studies, resulting in a total sum score on this sub-item of 25 out of a possible 28. With a sum score of 21, the next best scoring sub-item is “developers, sponsors and owners” which is also described partially or fully by all included studies. The sub-item “Quality assurance method” is described by none of the included studies. The sub-item “revisions and updates” is only partially described by three of the included studies.^44,47,50^ The sub-item “digital preservation” is only described by one included study.^44^

### Reported effects of the included interventions (RQ3)

Five out of the seven studies using a control or usual care group showed significantly better health results such as improved physical functioning, reduced anxiety, and reduced pain for the intervention group.^47,50–52,55^ The RCT from Strøm, Nielsen, et al. (2019) showed no effect on symptoms of anxiety and depression or patient-reported outcome, but the high compliance and degree of interaction with the digital health intervention suggested that it could be applicable in this group of patients. Venkatraman et al., 2022 found that the odds of having 90-day ER visits for app users were 32% lower than those for nonusers, but this difference was not statistically significant.

Most of the seven remaining studies without a control group showed promising results.^16,43–46,49,53^ Felbaum et al. (2018) reported almost all patients used the app and complied with instructions. They reported no canceled surgeries, one postoperative complication, and no readmissions for the patients in their study. Gautschi et al. (2010) found high satisfaction scores (>82%) with the intervention. Even though the app users in the study of Glauser et al. (2019) were also satisfied with the intervention, only 8 out of the 30 participants in the study were considered actual app users. Goz et al. (2019) reported the intervention was considered helpful by almost all patients, had a high degree of patient engagement and made the majority of patients less likely to call the clinic. Similarly, Ponder et al. (2020) reported that the majority of the respondents stated that the intervention was helpful during the pre- and postoperative period and 96% of them would recommend the app to friends or family. Some studies explored potential predictors of the use of digital health interventions. For example, Strøm, Hoybye, et al. (2019) showed that sociodemographic characteristics were not predictors of use of the digital health intervention, but women were more prone to be active users than men. Von Glinski et al. (2020) found that patient age, gender, or invasiveness of the surgery were not associated with usage of the digital health intervention.

### Methodological quality of included studies (RQ3)

The methodological quality of the included studies was reasonably high, with scores ranging from 24 to 35, out of a possible total score of 36 (see Table 5). The study of Venkatraman et al. (2022) was of the highest quality, with a score of 35 out of 36. This study scored fair on the item of “transferability and generalizability,” while scoring good on all the other items of the checklist. The studies of Erdogan (2020), Guo (2020), Hou (2019), Strøm, Hoybye, et al. (2019) and Strøm, Nielsen et al. (2019) were of the second highest quality, with a score of 34 out of 36. All these five studies scored good on the first seven items of the checklist and fair on the items “transferability and generalizability” and “implications and usefulness.”

**Table 5.** Quality assessment of methodology of included studies.

	1. Abstract and title	2. Introduction and aims	3. Method and data	4. Sampling	5. Data analysis	6. Ethics and bias	7. Results	8. Transferability or generalizability	9. Implications and usefulness	Total sum score per study (min. 9–max. 36)
Erdogan, 2020	Good 4	Good ` 4	Good 4	Good 4	Good 4	Good 4	Good 4	Fair 3	Fair 3	34
Felbaum, 2018	Fair 3	Good 4	Fair 3	Good 4	Good 4	Good 4	Good 4	Fair 3	Fair 3	32
Gautschi, 2010	Fair 3	Poor 2	Fair 3	Fair 3	Good 4	Fair 3	Fair 3	Fair 3	Fair 3	27
Glauser, 2019	Fair 3	Fair 3	Poor 2	Fair 3	Poor 2	Fair 3	Fair 3	Poor 2	Fair 3	24
Goz, 2019	Fair 3	Good 4	Fair 3	Good 4	Fair 3	Fair 3	Fair 3	Fair 3	Fair 3	29
Guo, 2020	Good 4	Good 4	Good 4	Good 4	Good 4	Good 4	Good 4	Fair 3	Fair 3	34
He, 2020	Good 4	Fair 3	Good 4	Good 4	Good 4	Fair 3	Good 4	Poor 2	Fair 3	31
Hou, 2019	Good 4	Good 4	Good 4	Good 4	Good 4	Good 4	Good 4	Fair 3	Fair 3	34
Ponder, 2020	Good 4	Fair 3	Fair 3	Good 4	Good 4	Good 4	Good 4	Fair 3	Good 4	33
Stewart, 2019	Fair 3	Fair 3	Fair 3	Good 4	Fair 3	Good 4	Good 4	Fair 3	Fair 3	30
Strøm, Hoybye, 2019	Good 4	Good 4	Good 4	Good 4	Good 4	Good 4	Good 4	Fair 3	Fair 3	34
Strøm, Nielsen, 2019	Good 4	Good 4	Good 4	Good 4	Good 4	Good 4	Good 4	Fair 3	Fair 3	34
Venkatraman, 2022	Good 4	Good 4	Good 4	Good 4	Good 4	Good 4	Good 4	Fair 3	Good 4	35
Von Glinski, 2020	Fair 3	Good 4	Fair 3	Fair 3	Good 4	Good 4	Fair 3	Fair 3	Poor 2	29
Total sum score per item (Min. 14, max. 56)	50	50	48	53	52	52	52	40	43	

The item “Sampling” was best described by the included studies (11 studies scoring good, 3 studies scoring fair, no studies scoring poor or very poor). The items “data analysis,” “ethics and bias” and “results” also scored high with a sum score of all the included studies of 52 out of a possible 56.

The items “transferability and generalizability” and “implications and usefulness” were least described in the studies: almost all studies scored fair or poor on these items, with only two studies with a “good” score.

## Discussion

The objective of the current scoping review was to provide a systematic overview of digital health interventions for spinal surgery patients described in scientific literature. The focus of this review was on describing the current digital health interventions, assessing the quality of these descriptions, and reviewing the reported effects in the included studies.

The first aim was to describe the current state of the art of digital health interventions for spinal surgery patients. A comprehensive, systematic search of the scientific literature yielded 14 full-text articles, describing 11 unique digital health interventions. Most studies were conducted in recent years demonstrating that digital health interventions for spinal surgery patients is a relatively new topic and emergent focus for scientific study. The identified digital health interventions differed in type of technology from a website, app, and web-based information system to a mobile phone messaging system and mobile phone interface. Most digital health interventions could be used before as well as after surgery. This is in line with findings in current research showing the wish of orthopedic patients to be granted a sense of control and responsibility over their recovery by initiating and using interventions preoperatively.^56^ This wish for a sense of control might also explain the high satisfaction scores mentioned in the included studies (e.g.^31,43,44,46^ Robinson et al. (2020) used the term surgical teachable moments to emphasize the potential of captivating the preoperative patient mind-set in encouraging perioperative behavior change and optimizing postoperative outcomes.^12^ A timely start of using digital interventions has also been underscored by Scott et al. (2017) who found that preoperative introduction of an intervention was superior and led to higher app use than an intervention with merely a postoperative timing.^57^ Our findings showed that most interventions focused on improving physical outcomes such as reducing pain and physical disability, while promoting the motivation and self-efficacy to do physical exercises. Other digital interventions focused on monitoring compliance with preoperative instructions and rehabilitation instructions. Only a few studies primarily focused on improving mental functioning. For example, the s-SPINA intervention^54^ contained animated information, an internet support group, and a diary to diminish depression and anxiety among patients. This lack of digital interventions focusing on the psychological functioning of spinal surgery patients is noteworthy as there is growing evidence that pre- and postoperative mindsets are related to long-term recovery and functioning.^58,59^ For example, psychological flexibility refers to the ability to adapt to challenging circumstances. In the context of pre- or postoperative pain, this means that painful sensations, feelings, and thoughts are accepted as opposed to avoided and that attention is shifted from pain avoidance towards personally valued goals.^60^ The concept of psychological flexibility is the main focus of Acceptance & Commitment Therapy, a relatively new form of behavioral therapy, found to be effective in treating chronic pain patients.^61,62^ In our introduction, we discussed the importance of a multidisciplinary approach to LBP and the complex interaction of multiple factors in the biopsychosocial model of (low back) pain. In this light, one would expect to find a large number of digital health interventions for spinal surgery patients focusing on psychological factors, but this was not the case in our review.

The second aim was to assess the quality of the description of the digital interventions for spinal surgery patients. For this assessment, we used the CONSORT eHealth checklist,^36^ a comprehensive and well-designed checklist for descriptions of digital health interventions. Our findings show that the four essential items in the checklist (access; mode of delivery, content of intervention, and theoretical framework; prompts and reminders; co-interventions) were only partially described in most studies. For example, a lack of use and description of theoretical frameworks is critical as this prevents understanding of and possible conclusions to be drawn on working mechanisms of the digital health intervention. Moreover, it has been shown that prompts and reminders positively influence the effectiveness of digital health interventions.^63^ Therefore, is it imperative to discuss whether or not prompts and reminders have been used in any digital health intervention. Seven items of the CONSORT eHealth checklist are highly recommended. Our findings revealed that some of these items such as “level of human involvement” and “developers, sponsors and owners” were generally adequately described in the studies. Other items such as “quality assurance method” and “digital preservation” were hardly described or not at all. Overall, these findings show a lack of systematic and comprehensive description of digital interventions. Unfortunately, this conclusion is in line with a recent systematic review on mobile devices and wearable technology for measuring patient outcomes after surgery.^59^ This finding is problematic, as, without this description, we cannot conclude what made an intervention effective or not. This seriously hampers our understanding of the possible working mechanisms of interventions and our ability to develop new interventions, based on earlier work.

The third aim of this scoping review was to explore potential effects reported in the included studies, including an assessment of their methodological quality. Many studies evaluated the acceptance of or satisfaction with the digital intervention. In general, the results show that digital interventions were well accepted by spinal surgery patients. For example, Felbaum et al. (2018) reported that almost all included participants used the app and Ponder et al. (2020) reported that 96% of their participants would recommend the app to friends or family. This is in line with previous research stating that orthopedic patients are open to using a digital health intervention.^29,30^ Seven studies included in this review used a control group. Five of these studies reported significantly stronger health-related effects such as improved physical functioning, reduced pain and anxiety in comparison to the control group. Though the evidence is very preliminary these findings suggest that digital interventions can promote physical and mental health. To assess the methodological quality of the studies a tool developed by Hawker et al. (2002) was used, comprising the following items: abstract and title; introduction and aims; method and data; sampling; data analysis; ethics and bias; presentation of results; transferability and generalizability; implications and usefulness. Overall, it was found that the quality of the studies was adequate to high. The quality items “transferability and generalizability” and “implications and usefulness” were least described in the included studies. This finding suggests that reflection on the potential implementation of the interventions and the results has not been a major focus of the included studies.

Though the current review underscores the acceptance and potential benefits of digital health interventions for spinal surgery patients, further research examining the impact on physical and mental health is warranted. Only four RCT studies were included in the review and the majority of the included studies had small sample sizes. Moreover, the targeted outcomes were varied, ranging from physical health, patient education, organizational, and patient satisfaction to cost-effectiveness. Overall, this shows that the field of digital interventions for spinal surgery patients is in the early stages of development. Although most included studies show promising results and digital interventions seem to be well-accepted in this target group, it is still an open question where most added value can be reached: whether this is helping patients stick to their (physical) rehabilitation plans, supporting their mental health, reduce canceled appointments, or a combination thereof. Future research is warranted to investigate what a digital intervention should focus on and what elements it should contain to reach those goals. As a next step, more rigorous evaluation studies could be considered.

### Strengths and limitations

A strength of the current review is the application of a systematic approach in combination with a broad range concerning search terms and lack of filters used during the search. Another strength of the current study is the fact it describes both the methodological quality of the included studies as well as the quality of the intervention description in these studies which, to our best knowledge, has not been conducted before. A potential limitation of the current study is the focus on digital health interventions developed specifically for spinal surgery patients. Other digital health interventions, suitable for several types of surgery, are potentially also of use for spinal surgery patients but were not included in the study. Future research could focus on exploring the quality of more generic digital health interventions and whether they could be beneficial to spinal surgery patients. In addition, the limited number of studies did not allow a meta-analytic assessment of the effects across studies. When more RCTs have been conducted in the future a meta-analytic analysis is warranted.

## Conclusions

The findings of this scoping review demonstrate that digital interventions are generally well accepted by spinal surgery patients and that there is preliminary evidence that these interventions improve adhering to rehabilitation programs and instructions and improve physical health. However, the findings of this review also show some important gaps in this emerging field of study. First, there is a scarcity of studies on the effects of digital interventions primarily focusing on psychological functioning and mental health. Second, the current development and description of digital interventions are suboptimal. Essential components such as the theory of specific working mechanisms based on behavior change models and the application of persuasive technologies are often lacking. Third, though the quality of the current studies is adequate, few studies used a more rigorous design to assess the efficacy of digital interventions for spinal surgery patients and there is a lack of longer term outcome assessment. Fourth, there is a lack of information and focus on transferability and implementation of the results and interventions.

## Appendix: Used search strings

### Step 1. Scopus

Used search string without using any filters:

(TITLE-ABS-KEY (smartphone OR ehealth* OR “e-health*” OR “electronic health*” OR mhealth* OR “m-health*” OR “mobil* health*” OR “mobil* application*” OR “mobil* intervention*” OR telehealth* OR “tele-health*” OR telemedic* OR “tele-medic*” OR telemonitor* OR “tele-monitor*” OR “persuas* technolog*” OR “persuas* health* technolog*” OR “behavio* intervention* technolog*” OR “digital* behavio* change intervention*” OR “digital* intervention*” OR “behavio* change support system*” OR “therap* comput* assist*” OR “online therap*” OR “web-based” OR website)) AND (TITLE-ABS-KEY (surgery OR surgeries OR surg*)) AND (TITLE-ABS-KEY (spine OR spinal OR spin* OR lumbar OR lumb*))

### Step 2. Pubmed

Used search string in Pubmed without using any filters:

Search: (“Mobile Applications"[Mesh]) OR “Telemedicine"[Mesh] OR smartphone [tiab] OR ehealth [tiab] OR “e-health"[tiab] OR “electronic health"[tiab] OR mhealth [tiab] OR “m-health"[tiab] OR “mobile health"[tiab] OR “mobile application"[tiab] OR “mobile intervention"[tiab] OR telehealth [tiab] OR “tele-health"[tiab] OR telemedic [tiab] OR “tele-medic” [tiab] OR telemonitor [tiab] OR “tele-monitor"[tiab] OR “persuasive technology"[tiab] OR “persuasive health technology” [tiab] OR “behavior intervention technology” [tiab] OR “digital behavioral change intervention” [tiab] OR “digital intervention” [tiab] OR “behavior change support system” [tiab] OR “therapy computer assisted” [tiab] OR “online therapy” [tiab] OR “web-based” [tiab] OR website [tiab] AND (surgery [tiab] OR surgeries [tiab] OR operation [tiab]) AND (spine [tiab] OR spinal [tiab] OR lumbar [tiab] OR “Spine/surgery"[Mesh])

### Step 3. Psych INFO

Used search string in Psych INFO (EBSCO Host) without using any filters:

smartphone OR ehealth* OR “e-health*” OR “electronic health*” OR mhealth* OR “m-health*” OR “mobil* health*” OR “mobil* application*” OR “mobil* app” OR “mobil* intervention*” OR telehealth* OR “tele-health*” OR telemedic* OR “tele-medic*” OR telemonitor* OR “tele-monitor*” OR “persuas* technolog*” OR “persuas* health* technolog*” OR “behavio* intervention* technolog*” OR “digital* behavio* change intervention*” OR “digital* intervention*” OR “behavio* change support system*” OR “therap* comput* assist*” OR “online therap*” OR “web-based” OR website OR online

AND surg* OR operation*

AND spin* OR lumb*

### Step 4. CINAHL Complete (EBSCO Host)

Used string (same as used in PsychINFO) in CINAHL without using any filters:

smartphone OR ehealth* OR “e-health*” OR “electronic health*” OR mhealth* OR “m-health*” OR “mobil* health*” OR “mobil* application*” OR “mobil* app” OR “mobil* intervention*” OR telehealth* OR “tele-health*” OR telemedic* OR “tele-medic*” OR telemonitor* OR “tele-monitor*” OR “persuas* technolog*” OR “persuas* health* technolog*” OR “behavio* intervention* technolog*” OR “digital* behavio* change intervention*” OR “digital* intervention*” OR “behavio* change support system*” OR “therap* comput* assist*” OR “online therap*” OR “web-based” OR website OR online

AND surg* OR operation*

AND spin* OR lumb*

### Step 5. IEEE Xplore Digital database

Used search string in Advanced search, option Command Search, in IEEE Xplore without using any filters:

((smartphone OR ehealth OR “e-health” OR “electronic health” OR mhealth OR “m-health” OR “mobile health” OR “mobile app*” OR “mobile intervention” OR telehealth OR “tele-health” OR telemedicine OR “tele-medicine” OR telemonitoring OR “tele-monitoring” OR “persuasive technology” OR “persuasive health technology” OR “behavio* intervention technology” OR “digital behavio* change intervention” OR “digital intervention” OR “behavio* change support system” OR “therapy computer assisted” OR “online therapy” OR “web-based” OR website OR online)

AND (surgery OR surgeries OR operation OR operations)

AND (spine OR spinal OR lumbar))

### Step 6. Web of Science

Used search string in Web of Science:

TS = ((smartphone OR ehealth* OR “e-health*” OR “electronic health*” OR mhealth* OR “m-health*” OR “mobil* health*” OR “mobil* application*” OR “mobil* app” OR “mobil* intervention*” OR telehealth* OR “tele-health*” OR telemedic* OR “tele-medic*” OR telemonitor* OR “tele-monitor*” OR “persuas* technolog*” OR “persuas* health* technolog*” OR “behavio* intervention* technolog*” OR “digital* behavio* change intervention*” OR “digital* intervention*” OR “behavio* change support system*” OR “therap* comput* assist*” OR “online therap*” OR “web-based” OR website) AND (surg* OR operation*) AND (spin* OR lumb*))

This search string is used in the Advance search option with “Topic” as the only filter (see TS before the search string), which means only articles in which the title, abstract of keywords contained the words in the search string were displayed. The option “All Databases” was selected, to ensure as many results as possible.

*Databases = WOS, KJD, MEDLINE, RSCI, SCIELO Timespan = All years*

*Search language = Auto*
